# The association between new graphic health warning labels on tobacco products and attitudes toward smoking among south Korean adolescents: a national cross-sectional study

**DOI:** 10.1186/s12889-020-08638-0

**Published:** 2020-05-24

**Authors:** Ji-eun Hwang, Sung-il Cho

**Affiliations:** 1 Institute of Health and Environment, Seoul National University, 1 Gwanak-ro, Gwanak-gu, Seoul, 08826 Republic of Korea; 2 Department of Public Health Science, Graduate School of Public Health, Seoul National University, 1 Gwanak-ro, Gwanak-gu, Seoul, 08826 Republic of Korea

**Keywords:** Smoking, Tobacco, Graphic health warning labels, Adolescents, Education, Smoke-free

## Abstract

**Background:**

Graphic health warning labels (GHWLs) on tobacco products are more effective than text warnings for communicating the risk of smoking. The implementation of GHWLs can prevent adolescents from initiating smoking. Therefore, this study examined the association between GHWLs newly implemented on December 23, 2016, in South Korea and attitudes toward smoking among adolescents.

**Methods:**

This post-implementation cross-sectional analysis examined the responses of 62,276 students (31,624 boys and 30,652 girls) who participated in the 2017 Web-based Korean Youth Risk Behavior Survey, which was completed anonymously as a self-administered questionnaire by middle and high school students. Multinomial logistic regression was applied to explore the attitudes toward smoking among the youth (13–18 years old) who have been exposed to GHWLs in order to identify relationship of exposure to the GHWLs with smoking initiation and awareness of the danger of *smoking.*

**Results:**

Six months after implementation, 69.4% of adolescents reported having been exposed to GHWLs in the previous 30 days. Among those exposed to GHWLs both boys and girls in grade 7 were significantly more likely than grade 12 high school students to decide not to start smoking (boys: AOR = 3.96, 95% CI 3.31–4.75, *p* < 0.001; girls: AOR = 2.76, 95% CI 2.32–3.30, *p* < 0.001) and to think that smoking was dangerous to their health (boys: AOR = 3.01, 95% CI 2.52–3.58, *p* < 0.001; girls: AOR = 2.42, 95% CI 2.03–3.88, *p* < 0.001) after seeing GHWLs. These associations were greater for adolescents who had experienced smoking-prevention education or had been exposed to anti-tobacco advertisements. However, those who smoked, used e-cigarettes, or experienced secondhand smoking were significantly less likely to decide not to smoke and to view smoking as dangerous.

**Conclusions:**

To maintain the perception of the harm of tobacco from childhood through adolescence, the government should implement both comprehensive tobacco controls, including smoking-prevention education in schools, and measures to encourage a smoke-free environment in homes.

## Background

Political and social changes that go beyond the regulation of tobacco products have been proposed for the *tobacco endgame,* which is a strategy to denormalize tobacco use and permanently remove tobacco products from society after a certain time point or to reduce smoking prevalence to a small percentage of the total population or in all population groups [1]. Although there is a lack of national consensus on a strategy [1], there is agreement about the necessity of ending the tobacco epidemic and protecting future generations from tobacco products [2, 3]. To accomplish this goal, the nation must set a date for ending the tobacco epidemic and implement various measures that focus on the product, user, market, and supply chain. Efforts by both the government and the public to confront the tobacco industry are important [4].

However, because of the difficulties in achieving the tobacco endgame goal, the best initial approach would be to implement the Framework Convention on Tobacco Control (FCTC) fully. Whereas 181 countries have ratified the FCTC [5], others have opted not to participate. Also, the average implementation rate of the FCTC differs depending on which article in the treaty is being discussed [5]. Article 8 (protection from exposure to tobacco smoke) has been ratified by 88% of the participating countries compared to 17% for Article 17 (support for economically viable alternative activities) [5].

The measures in the FCTC are effective when they are carried out comprehensively [6, 7]. These measures interact to create a smoking cessation environment throughout society, increasing smoking cessation efforts and preventing initiation of smoking. The tobacco endgame is the key to creating a smoke-free generation [2, 3]; consequently, education, publicity, and policies for smoking prevention among children and adolescents are essential.

Many countries have implemented various tobacco control policies to prevent smoking among children and youth [8, 9]. Increases in the price of tobacco products, school-based smoking-prevention education, smoke-free legislation, and mass media campaigns can prevent the initiation of smoking and reduce its prevalence among adolescents [8, 9].

Graphic health warning labels (GHWLs) on tobacco products are more effective than text warnings for communicating the risk of smoking [10]. Recently, regulations have been tightened, and standardized (plain), advertisement-free packaging has been implemented [11]. The implementation of such packaging may prevent the initiation of smoking by adolescents [10, 12]. However, only about half of FCTC countries have implemented GHWLs [5], and some have implemented one or two GHWLs on only 50% of tobacco products [5]. Such warnings are less effective than warnings on all tobacco products. Indeed, the warning pictures should be changed periodically to avoid familiarity, and their effectiveness should be assessed continuously [13].

GHWLs were implemented in South Korea on December 23, 2016 [14]. The 10 themes of the GHWLs are lung cancer, throat cancer, oral cancer, heart disease, stroke, secondhand smoke, smoking during pregnancy, impotence, skin aging, and premature death from cigarettes. Warnings are also printed on the packaging of electronic cigarettes (e-cigarettes), chewing tobacco, water pipes, and snus.

However, although exposure to the warning was examined just after the policy was initiated to assess its immediate impact, the South Korean policy has not been systematically investigated since then. Because one of the purposes of implementing GHWLs was to prevent the initiation of smoking by adolescents, it is important to evaluate whether adolescent awareness and attitudes have changed since the implementation. Studies in South Korea have found that text warnings on tobacco products are not effective in encouraging smoking cessation by current smokers [15, 16].

Therefore, this study investigated South Korean adolescents’ exposure to, and the effect of, GHWLs. First, demographic data about the survey participants and South Korean adolescents in general were gathered. Then, participants’ exposure to GHWLs during the previous 30 days, i.e., just after the policy was implemented, was determined, controlling for demographic characteristics associated with adolescents’ smoking, and the perceived effectiveness of GHWLs was assessed, including participants’ awareness of the harm of smoking and their intention to abstain from smoking after exposure to GHWLs. Sex differences in attitudes toward smoking were of particular interest [17–19]. Based on the results, a strategy for GHWLs and other tobacco-control policies targeted at adolescents are considered.

## Methods

### Participants

Data for this study were obtained from the 2017 Korea Youth Risk Behavior Web-based Survey (KYRBWS), which is conducted jointly by the Korean Ministry of Education, the Korean Ministry of Health and Welfare, and the Korean Center for Disease Control and Prevention to monitor the health behavior of South Korean adolescents [20]. The KYRBWS, a national cross-sectional survey [21], targeted middle and high school students in grades 7 to 12 in all regions of South Korea, including 5632 schools and 3,027,488 students [20]. To obtain a representative sample of students, the KYRBWS applied a complex sampling design including stratification, clustering, and multistage probability sampling [21]. All students in one class at 400 selected middle schools and 400 high schools participated in the survey [20]. In 2017, the survey was conducted from May to June, and the response rate was 95.8% [20]. In total, 62,276 students participated in the 2017 KYRBWS [20]; the present study analyzed data from all of them.

### Measurements

The 2017 KYRBWS was conducted anonymously in a computer room, where each student completed a self-administered, Web-based questionnaire consisting of 123 questions [20].

#### Exposure to GHWLs during the past 30 days

To assess their exposure to GHWLs, participants were asked the following: “For the past 30 days, did you see a graphic health warning on tobacco products?” The responses were classified as (1) No and (2) Yes.

#### Perceived effectiveness of GHWLs after having viewed a warning

This study used two questions from the Korea Youth Risk Behavior Web-based Survey. Participants who responded that they saw GHWLs in past 30 days were asked two questions: “When you saw the graphic health warning, did you think that you should not smoke?” and “When you saw the graphic health warning, did you think that smoking is harmful to health?”. The responses to these questions were classified as (1) not at all, (2) slightly, (3) very, or (4) extremely. These questions are commonly used in the assessment of GHWLs and were taken from the peer-reviewed literature [22–25].

#### Smoking status

Cigarette-smoking status was assessed as the number of smoking days during the past 30 days. Respondents who reported smoking during > 1 day were classified as current smokers, whereas those who reported smoking 0 days were classified as non-smokers. Respondents’ e-cigarette smoking status was assessed by the number of days on which they used an e-cigarette during the past 30 days. Respondents who reported > 1 days of e-cigarette use were classified as current users of e-cigarettes; respondents who reported 0 days of e-cigarette use were classified as non-users of e-cigarettes.

#### Other indicators related to smoking

Students were classified as exposed to secondhand smoke if someone in their household smoked during the previous 7 days. Students were considered to have experienced smoking-prevention education in school if they attended such a program during class time during the previous 12 months or if they were exposed to relevant educational broadcasts, participated in large group programs, and so on. Experience with anti-tobacco advertisements was defined as having seen or heard publicity about tobacco control during the previous 12 months (e.g., TV, radio, news, the Internet, newspapers, subways and bus stops, etc.). Exposure to tobacco advertisements was defined as having seen any tobacco advertisements in magazines, convenience stores, or supermarkets or on the Internet during the last 30 days.

#### Covariates

The analysis was controlled for the following demographic characteristics: gender, grade, educational stage, region, family living structure, perceived household economic status, perceived academic record, perceived stress, experience of depression, and current alcohol use. Regions were divided into metropolitan, medium and small cities, and rural. Family living structures were divided into three levels according to the current parental residence: living with both parents, living with a single parent, and other (living with grandparent, living with brother or sister, or living alone). Perceived household economic status was evaluated by the following question: “How would you rate your family’s economic status?” The responses were re-categorized into three levels: wealthy (very wealthy or wealthy), medium income, and poor (poor or very poor). Perceived academic record was evaluated by the following question: “How would you describe your school grades?” Responses were re-categorized in three levels: high (high and upper middle), middle, and low (lower middle and low).

Perceived stress was assessed by asking the following: “How much stress are you experiencing in your daily life?” The possible responses were (1) a very high level, (2) a high level, (3) a moderate level, (4) a low level, and (5) none. Experience of depression was assessed by asking the following: “In the past 12 months, have you experienced sadness or despair that interrupted your everyday life for a period of 2 weeks?” The responses were (1) Yes or (2) No. Current alcohol use was assessed as the number of drinking days during the past 30 days. Respondents who reported > 1 days of drinking were classified as current alcohol users, whereas those reporting 0 days of drinking were classified as non-drinkers.

### Data analyses

We conducted a statistical analysis of the KYRBWS that reflected the weights assigned considering the complex sampling survey design. A frequency analysis was conducted to assess the number and percentage of subjects according to demographic characteristics. The distribution of adolescents who had seen GHWLs in the past 30 days varied by gender and demographic characteristics. In addition, multinomial logistic regression was applied to explore the relationship between GHWLs and perceived effectiveness among students who had seen GHWLs on tobacco products in the past 30 days. In this test, the following covariates were adjusted for region, family living structure, perceived academic record, perceived household economic status, perceived stress, experience of depression, and current alcohol use; they are presented as adjusted odds ratios (AORs) and 95% confidence intervals (CIs). All statistical analyses were stratified by gender. In addition, the data analyses were performed using SPSS ver. 24, and *p*-values < 0.05 were considered indicative of significance.

## Results

### Demographic characteristics

Approximately half (51.4% in total; 51.3% of boys and 51.5% of girls) of the adolescents currently live in metropolitan areas, and the majority live with both parents (83.9% in total; 83.5% of boys and 84.2% of girls) (Table 1). In addition, more than one-third evaluated their household economic level as medium (45.6% in total; 42.9% of boys and 48.4% of girls) and their academic record as high (39.2% in total; 40.8% of boys and 37.3% of girls). Most students reported stress in their lives (High 37.2% vs. Intermediate 42.6 vs. Low 20.2%), and 25.1% of all students had experienced depression (20.3% of boys and 30.3% of girls). Of the students, 16.1% (18.2% of boys and 13.2% of girls) were current alcohol users.

**Table 1:** Demographic characteristics of the survey participants (*n* = 62,276)

Variable	Total (*n* = 62,276)		Boys (*n* = 31,624)		Girls (*n* = 30,652)	
	n	(%)^a^	n	(%)^a^	n	(%)^a^
Grade						
7th grade	10,189	(14.8)	5178	(14.8)	5011	(14.9)
8th grade	10,377	(15.4)	5272	(15.3)	5105	(15.4)
9th grade	10,319	(15.1)	5202	(15.1)	5117	(15.0)
10th grade	10,165	(17.1)	5069	(17.1)	5096	(17.1)
11th grade	10,800	(19.0)	5610	(19.1)	5190	(18.9)
12th grade	10,426	(18.6)	5293	(18.6)	5133	(18.7)
Educational stage						
Middle school	30,885	(45.3)	15,652	(45.2)	15,233	(45.3)
High school	31,391	(54.7)	15,972	(54.8)	15,419	(54.7)
Region						
Metropolitan	32,065	(51.4)	15,848	(51.3)	16,217	(51.5)
Medium and small cities	26,614	(44.0)	13,940	(44.2)	12,674	(43.9)
Rural	3597	(4.6)	1836	(4.5)	1761	(4.6)
Family living structure						
Living with both parents	51,735	(83.9)	26,176	(83.5)	25,559	(84.2)
Living with a single parent	8729	(13.5)	4440	(13.5)	4289	(13.4)
Others	1812	(2.6)	1008	(3.0)	804	(2.4)
Perceived household economic status						
Wealthy	24,802	(40.4)	13,618	(43.4)	11,184	(37.1)
Medium	28,582	(45.6)	13,664	(42.9)	14,918	(48.4)
Poor	8892	(14.0)	4342	(13.7)	4550	(14.5)
Perceived academic record						
High	24,524	(39.2)	12,925	(40.8)	11,599	(37.3)
Middle	17,810	(28.7)	8683	(27.6)	9127	(30.0)
Low	19,942	(32.1)	10,016	(31.6)	9926	(32.7)
Perceived stress						
High	23,259	(37.2)	9552	(30.4)	13,707	(44.6)
Middle	26,271	(42.6)	13,745	(43.9)	12,526	(41.2)
Low	12,746	(20.2)	8327	(25.8)	4419	(14.2)
Depression experience						
Yes	15,612	(25.1)	6326	(20.3)	9286	(30.3)
No	46,664	(74.9)	25,298	(79.7)	21,366	(69.7)
Current alcohol use						
User	9597	(16.1)	5562	(18.2)	4035	(13.2)
Non-user	52,679	(83.9)	26,062	(81.8)	26,617	(86.8)

^a^Weighted percentages considering the complex sample survey design

### Exposure to GHWLs during the past 30 days

More than two-thirds of the respondents (69.4%; 66.6% of boys and 72.4% of girls) reported that they had been exposed to GHWLs during the past 30 days. In boys, such exposure was most frequent among tenth graders; in girls, it was most frequent among ninth graders (Table 2). Current smokers (85.4% of boys and 87.7% of girls) and users of e-cigarettes (87.5% of boys and 84.3% of girls) were more likely to be exposed to GHWLs than were non-smokers (64.7% of boys and 71.9% of girls) and non-users (65.9% of boys and 72.3% of girls). Additionally, those who were exposed to secondhand smoke (boys: 75.6% vs. 63.3%; girls: 80.7% vs. 69.0%), tobacco advertisements (boys: 71.5% vs. 49.1%; girls: 77.5% vs. 53.2%), or anti-tobacco advertisements (boys: 71.8% vs. 47.1%; girls: 75.6% vs. 51.8%) were more likely to have been exposed to GHWLs than those who were not. In the past 12 months, 74.0% of boys and 76.5% of girls who had been educated about smoking prevention at school were exposed to GHWLs compared to only 50.6% of boys and 61.2% of girls who had not received such education.

**Table 2:** Prevalence of exposure to graphic health warnings on tobacco products for the past 30 days among South Korean adolescents (*n* = 62,276)

Variable	Boys (*n* = 31,624)				Girls (*n* = 30,652)			
	Yes		No		Yes		No	
	n	(%)^a^	n	(%)^a^	n	(%)^a^	n	(%)^a^
Grade								
7th grade	3425	(66.9)	1753	(33.1)	3599	(72.3)	1412	(27.7)
8th grade	3467	(66.0)	1805	(34.0)	3711	(72.9)	1394	(27.1)
9th grade	3401	(65.5)	1801	(34.5)	3749	(73.0)	1368	(27.0)
10th grade	3482	(68.6)	1587	(31.4)	3654	(71.6)	1442	(28.4)
11th grade	3703	(65.8)	1907	(34.2)	3746	(72.2)	1444	(27.8)
12th grade	3576	(67.0)	1717	(33.0)	3718	(72.3)	1415	(27.7)
Current smoking status								
Smoker	2437	(85.4)	421	(14.6)	789	(87.7)	122	(12.3)
Non-Smoker	18,617	(64.7)	10,149	(35.3)	21,388	(71.9)	8353	(28.1)
e-cigarette status								
User	864	(87.5)	127	(12.5)	214	(85.3)	39	(14.7)
Non-User	20,190	(65.9)	10,443	(34.1)	21,963	(72.3)	8436	(27.7)
Secondhand Smoke								
Non-exposed	14,407	(63.3)	8383	(36.7)	14,978	(69.0)	6743	(31.0)
Exposed	6647	(75.6)	2187	(24.4)	7199	(80.7)	1732	(19.3)
Tobacco Advertise								
Non-exposed	3361	(49.1)	3505	(50.9)	3408	(53.2)	2978	(46.8)
Exposed	17,693	(71.5)	7065	(28.5)	18,769	(77.5)	5497	(22.5)
Anti-Tobacco Advertisement								
Non-exposed	3055	(47.1)	3455	(52.9)	2141	(51.8)	1984	(48.2)
Exposed	17,999	(71.8)	7115	(28.2)	20,036	(75.6)	6491	(24.4)
Experience of smoking prevention education in school								
Yes	16,218	(74.0)	5806	(26.0)	17,379	(76.5)	5395	(23.5)
No	4836	(50.6)	4764	(49.4)	4798	(61.2)	3080	(38.8)

^a^Weighted percentages considering the complex sample survey design

### Thinking about the harm of smoking after seeing a GHWL

Table 3 shows the results of a multinomial logistic regression analysis (after adjustment for all covariates) of the relationship between perception of the harm of smoking and other variables among boys and girls who had seen GHWLs in the 30 days prior to the survey. The probability of thinking about smoking harm after exposure to GHWLs was higher among boys and girls in the lower grades than among those in the higher grades (seventh-grade boys: AOR 3.96, 95% CI 3.31–4.75; seventh-grade girls: AOR 2.76, 95% CI 2.32–0.30). The probability of thinking about the harm of smoking was significantly lower in smokers (boys: AOR 0.25, 95% CI 0.23–0.28; girls: AOR 0.25, 95% CI 0.21–0.30) than in non-smokers. Users of e-cigarettes (boys: AOR 0.85, 95% CI 0.72–0.99; girls: AOR 0.74, 95% CI 0.53–1.05) were also less likely than non-users to think of the harm of smoking. Those who had received education regarding smoking prevention during school during the last 12 months were more likely to think about smoking-related harm compared to those who did not receive smoking-prevention education in school (boys: AOR 1.38, 95% CI 1.26–1.50; girls: AOR 1.20, 95% CI 1.10–1.32). Furthermore, the AOR for thinking about smoking harm was 1.48 (95% CI 1.35–1.62) for boys and 1.85 (95% CI 1.64–2.08) for girls who had been exposed to anti-tobacco advertisements during the past 12 months compared to those not exposed to such advertising.

**Table 3:** The relationship between perception about the harm of smoking and other variables after exposure to graphic health warnings during the previous 30 days by South Korean boys and girls

Variable	Boys				Girls			
	N	(%)^a^	AOR^b^	(95% CI)	n	(%)^a^	AOR^b^	(95% CI)
Grade								
7th grade	3242	(94.6)	**3.96**	(3.31–4.75)	3390	(94.0)	**2.76**	(2.32–3.30)
8th grade	3082	(88.3)	**1.93**	(1.67–2.23)	3356	(90.0)	**1.77**	(1.52–2.06)
9th grade	2899	(85.0)	**1.63**	(1.41–1.88)	3337	(89.0)	**1.65**	(1.43–1.90)
10th grade	2726	(78.0)	**1.16**	(1.02–1.31)	3165	(86.4)	**1.42**	(1.25–1.60)
11th grade	2773	(74.2)	1.10	(0.98–1.23)	3110	(83.1)	**1.16**	(1.02–1.31)
12th grade	2546	(70.6)	1.00		2990	(80.2)	1.00	
Current smoking status								
Smoker	1180	(47.7)	**0.25**	(0.23–0.28)	404	(52.4)	**0.25**	(0.21–0.30)
Non-Smoker	16,088	(85.6)	1.00		18,944	(88.1)	1.00	
e-cigarette status								
User	427	(49.7)	**0.85**	(0.72–0.99)	108	(52.1)	0.74	(0.53–1.05)
Non-User	16,841	(82.4)	1.00		19,240	(87.1)	1.00	
Expose to Secondhand Smoke								
Exposed	5317	(78.9)	0.97	(0.90–1.05)	6174	(85.4)	0.99	(0.90–1.09)
Non-exposed	11,951	(81.9)	1.00		13,174	(87.4)	1.00	
Tobacco Advertise								
Exposed	14,627	(81.6)	1.09	(0.98–1.22)	16,453	(87.3)	1.11	(0.99–1.24)
Non-exposed	2641	(77.6)	1.00		2895	(83.8)	1.00	
Anti-Tobacco Advertisement								
Exposed	15,057	(82.7)	**1.48**	(1.35–1.62)	17,699	(87.9)	**1.85**	(1.64–2.08)
Non-exposed	2211	(71.2)	1.00		1649	(76.3)	1.00	
Experience of smoking prevention education in school								
Yes	13,580	(82.9)	**1.38**	(1.26–1.50)	15,325	(87.8)	**1.20**	(1.10–1.32)
No	3688	(74.9)	1.00		4023	(83.3)	1.00	

*AOR* Adjusted odds ratio, *CI* Confidence interval

^a^Weighted percentages considering the complex sample survey design

^b^Obtained from multinomial logistic regression analysis with control variables; Region, Family living structure, Perceived academic record, Perceived household economic status, Perceived stress, Depression experience, Current alcohol use

### Intention not to start smoking after having seen GHWLs

The results of the logistic regression analysis of the relationship between the intention not to start smoking after exposure to GHWLs during the previous 30 days and smoking-related variables, controlling for all covariates, are shown in Table 4. As with thinking about the harm of smoking, the intention not to start smoking was associated with grade, current smoking status, use of e-cigarettes, smoking-prevention education in school, and exposure to anti-tobacco advertisements between both genders. Also, boys who had been exposed to secondhand smoke during the past 7 days were significantly less likely to report the intention to start smoking than were boys (boys: AOR 0.85, 95% CI 0.79–0.92; girls: AOR 0.96, 95% CI 0.88–1.05) not exposed to secondhand smoke. The AOR was 1.03 (95% CI 0.92–1.15) for boys and 1.22 (95% CI 1.10–1.35) for girls who had been exposed to tobacco advertisements during past 30 days, but this was significant only in girls.

**Table 4:** The relationship between the intention not to start smoking and other variables after exposure to graphic health warnings during the past 30 days among South Korean boys and girls

Variable	Boys				Girls			
	N	(%)^a^	AOR^b^	(95% CI)	n	(%)^a^	AOR^b^	(95% CI)
Grade								
7th grade	3190	(93.2)	**3.01**	**(2.52–3.58)**	3393	(94.3)	**2.42**	**(2.03–2.88)**
8th grade	3041	(87.1)	**1.78**	**(1.56–2.04)**	3358	(90.1)	**1.58**	**(1.36–1.84)**
9th grade	2850	(83.6)	**1.58**	**(1.37–1.82)**	3334	(88.8)	**1.49**	**(1.29–1.73)**
10th grade	2693	(77.2)	**1.23**	**(1.09–1.40)**	3180	(87.0)	**1.43**	**(1.25–1.63)**
11th grade	2676	(71.5)	**1.11**	**(1.00–1.24)**	3147	(84.1)	**1.26**	**(1.11–1.43)**
12th grade	2431	(67.3)	1.00		2985	(80.4)	1.00	
Current smoking status								
Smoker	817	(32.1)	**0.15**	**(0.13–0.16)**	264	(33.6)	**0.13**	**(0.11–0.16)**
Non-Smoker	16,064	(85.6)	1.00		19,133	(89.1)	1.00	
e-cigarette status								
User	285	(32.5)	**0.64**	**(0.53–0.78)**	63	(29.4)	**0.50**	**(0.32–0.78)**
Non-User	16,596	(81.2)	1.00		19,334	(87.7)	1.00	
Expose to Secondhand Smoke								
Exposed	5073	(74.8)	**0.85**	**(0.79–0.92)**	6127	(84.7)	0.96	(0.88–1.05)
Non-exposed	11,808	(81.1)	1.00		13,270	(88.2)	1.00	
Tobacco Advertise								
Exposed	14,272	(79.5)	1.03	(0.92–1.15)	16,507	(87.6)	**1.22**	**(1.10–1.35)**
Non-exposed	2609	(76.9)	1.00		2890	(83.9)	1.00	
Anti-Tobacco Advertisement								
Exposed	14,701	(80.7)	**1.34**	**(1.23–1.47)**	17,710	(88.0)	**1.55**	**(1.35–1.77)**
Non-exposed	2180	(70.2)	1.00		1687	(78.4)	1.00	
Experience of smoking prevention education in school								
Yes	13,267	(81.0)	**1.40**	**(1.28–1.53)**	15,361	(88.0)	**1.23**	**(1.12–1.36)**
No	3614	(73.2)	1.00		4036	(83.8)	1.00	

*AOR* Adjusted odds ratio, *CI* Confidence interval

^a^Weighted percentages considering the complex sample survey design

^b^Obtained from multinomial logistic regression analysis with control variables; Region, Family living structure, Perceived academic record, Perceived household economic status, Perceived stress, Depression experience, Current alcohol use

## Discussion

We examined whether adolescents had been exposed to GHWLs and whether such exposure was associated with their perception of smoking. The results show that most youth had been exposed to GHWLs during the previous month and that such exposure had enhanced their intention not to start smoking. The GHWLs also effectively communicated the risks of smoking. Exposure to GHWLs was most strongly associated with prior experience with other smoking-prevention education or anti-tobacco advertisements. Furthermore, current smokers, current users of e-cigarettes, and those exposed to secondhand smoke were less associated by the GHWLs than were those who had not had such experiences.

As young people progress in school, the probability that they will consider smoking to be harmful to health is reduced, as is the likelihood that they will not consider starting to smoke. Just as the increase in age or grade is strongly associated with smoking initiation in adolescents [26], risky behaviors, such as drinking alcohol and smoking, also peak as a function of aging [27]. Because youth are prone to engage in risky behavior during adolescence, it will be difficult to prevent smoking in this population using GHWLs alone [28]. To prevent smoking, it is important that a well-formed negative attitude toward smoking continue from childhood into adolescence so that this same attitude can be carried into adulthood [29, 30]. For this reason, young people should be continually educated, starting in childhood, about the harm of smoking and the benefits of not smoking; such efforts will eliminate curiosity about smoking and help youth resist the temptation to start smoking.

However, young people who already smoked were less associated by the content of the GHWLs, and they were less likely to see smoking as harmful than were non-smokers who saw the warnings. This may be due to cognitive dissonance and defensiveness among those who already smoked [31–34]. Changing the attitudes and awareness of young smokers requires a more persuasive warning. Adolescent smokers prefer loss- rather than gain-framed warnings [35], and the guilt and fear arising from such warnings tend to reinforce their impact [36]. Additionally, an environment in which curiosity-inducing factors are blocked beforehand is required, as is a continued understanding of why adolescents smoke.

Smoking susceptibility is an important predictor of smoking in adolescents. The susceptibility to smoking is related to several variables, including genetic (e.g., race, ethnicity) [37], environmental (e.g., exposure to tobacco advertisements) [38], and psychosocial and lifestyle (e.g., aggression, depression, anxiety, sensation-seeking) factors [39, 40]. In particular, exposure to secondhand smoke not only adversely affects the health of infants and young children [41] but also increases their likelihood of smoking [42]. Indeed, secondhand smoke exposure is associated with a greater likelihood of being a smoker and of initiating smoking [42].

Although significant only in boys, exposure to secondhand smoke during the past 7 days was associated with a reduced intention not to start smoking in this study. Continuous exposure to secondhand smoke may lead to underestimation of the risk of using tobacco products and to familiarity with them. As a result, GHWLs alone are insufficient to change the perceptions and attitudes of tobacco-friendly adolescents [43], such as current smokers who are familiar with tobacco products and those exposed to secondhand smoke. These results suggest that a single policy is inadequate for the prevention of smoking by adolescents and underscore the need for multiple integrated approaches.

Smoking-prevention education for students is not limited to simply informing them about the risk of smoking; it can also help to strengthen the negative opinions about smoking, thus preventing smoking initiation [44]. In this study, educated adolescents were more aware of the health consequences of the warning picture than were those who were not educated, and the hesitancy of the former group to start smoking was strengthened. Thus, smoking-prevention education at school is important for tobacco control because it positively affects the impact of relevant policies [45, 46]. To maximize the effectiveness of smoking-prevention education, it should be coordinated with other tobacco-control policies to prevent smoking among adolescents [45, 46].

Anti-tobacco advertisements are also part of tobacco-control policy and are aimed at preventing smoking initiation in youth. However, among teenagers, GHWLs led to a greater health awareness and stronger non-smoking intent than did anti-tobacco advertisements alone. Because promotion of smoking cessation or prevention of smoking is more effective when used in combination with other methods, multiple approaches should be implemented [47]. Therefore, tobacco-control policies and messages must be promoted to increase awareness of the effects of tobacco products and smoking and to promote negative attitudes toward them.

Our results have important implications for the tobacco endgame. The tobacco-free-generation approach, a key endgame strategy, is based on the goal of legally preventing individuals born after a certain year from accessing tobacco products [2, 3]. Public support for such a mandatory approach would be enhanced by tobacco denormalization, and plain packaging is a useful step toward this goal. Because denormalization strategies typically focus on tobacco use or tobacco users [2, 3], a plain-packaging policy has greater potential as it serves to denormalize tobacco products, thus justifying subsequent legal measures. However, our results suggest that denormalization of tobacco products may have limited efficacy in youth who have experienced active or passive smoking. Indeed, a focus on a particular cohort may not be effective, as extant attitudes and experiences may blunt the impact of such an approach. The logical follow up to tobacco product denormalization by means of packaging policy is denormalization of the tobacco industry, which justifies control of the supply chain. We suggest that future anti-smoking policies focus on denormalization of the tobacco industry.

Tobacco-packaging policy, whether strong or weak, removes the attraction of tobacco and reduces its value so that it is not worth purchasing. Currently, packaging policy concentrates on denormalizing the tobacco product by imparting information about its harmful effects. It is important to continue strengthening packaging policy so that the packaging of all tobacco products is plain. However, any packaging policy is only one of the various comprehensive measures required to denormalize the tobacco industry, to eradicate the primary force driving smoking [48]. Research on how the packaging contents impart this message, and the combinations of policies required to achieve denormalization of the tobacco industry, is needed.

This study has several limitations. First, the survey was performed from May to June 2017, a period that differed from that of the exposure to the GHWLs. Second, the exposure pathway of the GHWLs could not be determined precisely; possibilities include the media, points of sale, school curriculum, and/or cigarette packs. These different pathways of exposure may have affected the perceptions of and attitudes toward smoking and should be evaluated as part of future studies. Third, the students who had seen GHWLs were asked about their future intention not to start smoking, but this study did not compare their responses to those of youth who had not seen GHWLs; this should be the subject of future work. Finally, because this was a cross-sectional study, we examined the relationship between the implementation of new GHWLs and their perceived effectiveness on adolescents’ attitudes toward smoking; we could not assess the causal relationship between GHWLs and smoking attitudes. Also, since this study used secondary data, the researchers cannot be involved in selecting research instruments to measure the effect of GHWLs exposure. For long-term analysis, various instruments with validity and accuracy are needed for multilateral assessment of the effect of exposure to GHWLs*.* It is necessary to establish a long-term cohort study to examine whether GHWLs are effective in deterring adolescents from initiating smoking and, if so, what the degree of that effectiveness is.

## Conclusion

The GHWLs strengthened the perception of the risk of smoking and the intention not to start smoking among adolescents. The positive association of GHWLs with risk awareness was greater in adolescents who had experienced smoking-prevention education or who had been exposed to anti-tobacco advertisements. By contrast, the association decreased among those who were already smokers or users of e-cigarettes, as well as in students in higher grades and young people exposed to secondhand smoke at home. Comprehensive school-based smoking-prevention education and efforts to promote smoke-free home environments are needed to help adolescents carry their negative perceptions about smoking into adulthood.

## Data Availability

The microdata of KYRBS are released annually in December each year [21]. The datasets analyzed during the current study are available on the KYRBS website, http://yhs.cdc.go.kr.

## Abbreviations

FCTC: Framework Convention on Tobacco Control
GHWLs: Graphic Health Warning Labels
KYRBWS: Korea Youth Risk Behavior Web-based Survey
AORs: adjusted odds ratios
CIs: confidence intervals
